# Cytomegalovirus pneumonitis amid COVID-19 chaos: the hidden enemy—a case report

**DOI:** 10.1186/s13256-022-03259-0

**Published:** 2022-01-31

**Authors:** Abdulkarim Yousef Aldehaim, Abrar Mohammed Alfaifi, Seham Nehdal Hussain, Abdulaziz Mohammed Alrajhi

**Affiliations:** 1 Rheumatology Unit, Department of Medicine, King Saud University, Riyadh, Saudi Arabia; 2 Department of Medicine, King Saud University, Riyadh, Saudi Arabia; 3 Infectious Diseases Unit, Department of Medicine, King Saud University, Riyadh, Saudi Arabia

**Keywords:** COVID-19, SARS-CoV-2, Cytomegalovirus, CMV, Case report

## Abstract

**Background:**

The effect of coronavirus disease 2019 on the immune system is increasingly recognized. When severe, it causes immune dysregulation that may favor other infections, including Herpesviridae. Cytomegalovirus shares many innate immune pathways with severe acute respiratory syndrome coronavirus 2, which may potentiate each other. We describe a case of cytomegalovirus pneumonitis complicating the course of coronavirus disease 2019 in a patient with systemic lupus erythematosus/systemic sclerosis overlap and usual interstitial pneumonia, mimicking interstitial lung disease exacerbation. To the best of the authors’ knowledge, this is the first case to be reported worldwide in the setting of connective tissue disease-associated interstitial lung disease.

**Case description:**

We describe the case of a 47-year-old white/Yemeni female who is known to have systemic lupus erythematosus/scleroderma overlap and usual interstitial pneumonia who was initially admitted with severe coronavirus disease 2019 pneumonia mandating intensive care. After initial improvement, it was later complicated with cytomegalovirus pneumonitis, mimicking interstitial lung disease exacerbation. The case was successfully treated with ganciclovir.

**Conclusion:**

Intriguingly, severe acute respiratory syndrome coronavirus 2 and cytomegalovirus may potentiate each other, since they share some innate immune pathways. Subjects with severe coronavirus disease 2019 and underlying connective tissue diseases and those who are immunosuppressed carry higher risk compared with other cohorts, which may mandate active surveillance for cytomegalovirus coinfection or reactivation. Among various immunosuppressive therapies that has been tried for cytokine storm, use of anti-interleukin-6 inhibitors in the aforementioned population may carry more harm than previously thought, which may suggest that is reasonable to omit its use in treating this group with coronavirus disease 2019. This case underlines an underrecognized and underreported cause of morbidity and mortality during the course of severe coronavirus disease 2019 and will help to alert clinicians of its occurrence.

## Background

The World Health Organization (WHO) declared COVID-19 to be a public health emergency of international concern [1]. As of October 2020, COVID-19 had infected more than 37 million people worldwide, with more than a million deaths reported to the WHO [2]. As a new disease, many theories have been hypothesized about its pathogenesis and its variable clinical course including postinflammatory phenomena [3]. Thus, it may in turn hinder other mimickers.

The hallmark of the disease is lymphopenia, which negatively influences cell-mediated immunity [4]. A state of weakened cell-mediated immunity increases the risk of various viral infections, including Herpesviridae [5]. A recent case series of 38 patients with COVID-19 determined the pulmonary reactivation rate in herpes simplex virus (HSV) and cytomegalovirus (CMV). In this cohort including 38 patients with COVID-19, 9 had HSV reactivation, 2 had CMV reactivation, and 7 had coactivation [6]. This represent 47% of the sample, raising concerns that these occurrences are widely underreported and underrecognized, thus majorly contributing to increased morbidity and mortality.

We describe herein a case of CMV pneumonitis complicating the course of COVID-19 in a patient with systemic lupus erythematosus (SLE)/systemic sclerosis (SSC) overlap and usual interstitial pneumonia (UIP), mimicking interstitial lung disease (ILD) exacerbation. To the best of the authors’ knowledge, this is the first case to be reported worldwide in the setting of connective tissue disease (CTD)-associated interstitial lung disease (ILD).

## Case description

**Fig. 1:**
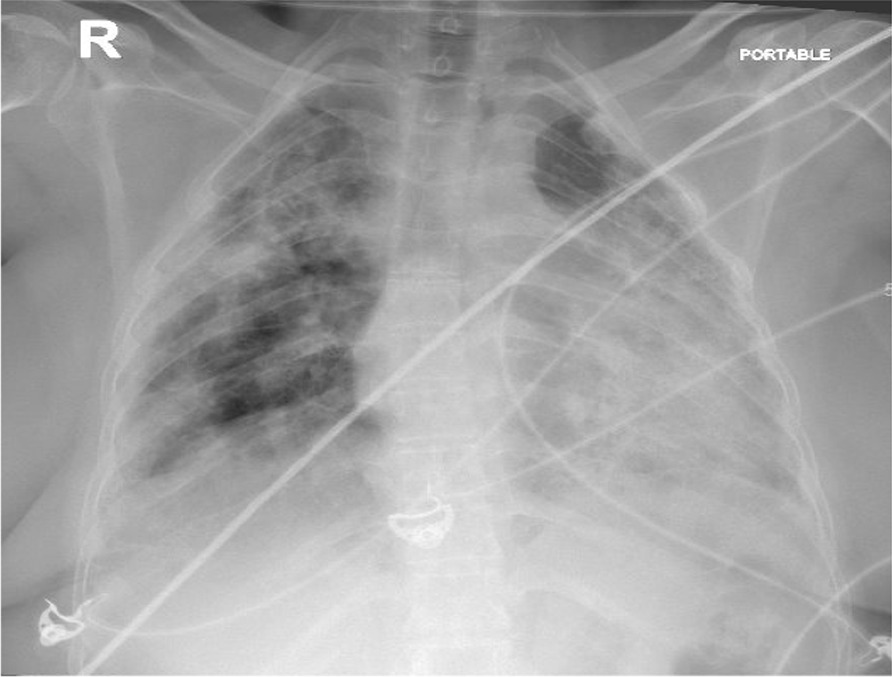
Chest X ray on day 1 of admission, showing extensive bilateral consolidations

**Fig. 2:**
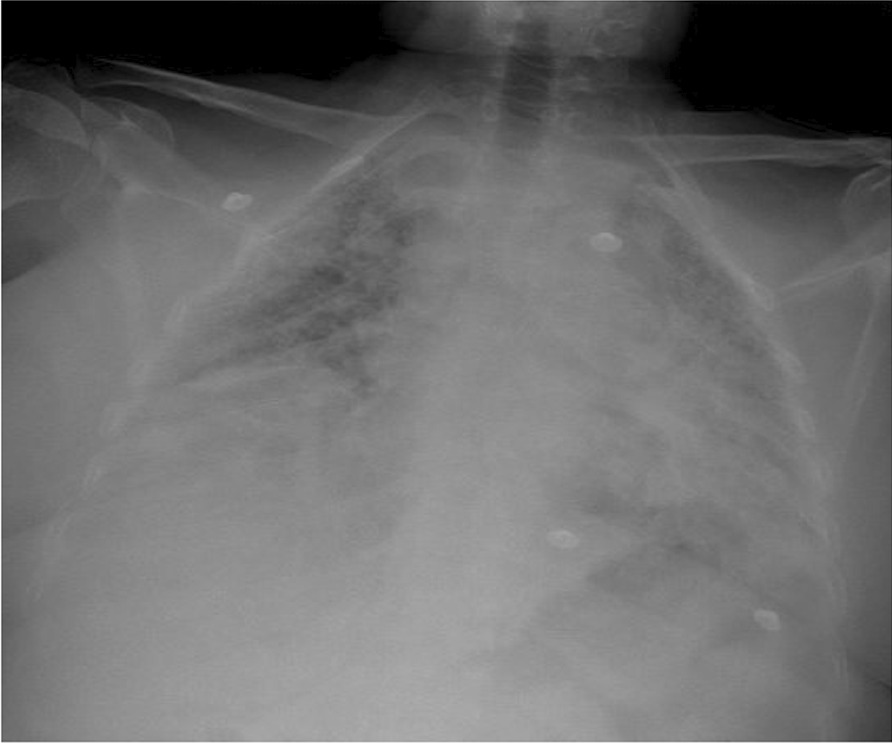
Repeated chest X-ray at day 21, showing worsening infiltrates

A 47-year-old white/Yemeni female presented with shortness of breath, productive cough, and fever for 5 days. She was known to have SLE/SSC overlap, UIP, and pulmonary hypertension. Her home medications included hydroxychloroquine 200 mg daily, mycophenolate mofetil 500 mg twice daily, prednisolone 5 mg daily, sildenafil 50 mg twice daily, and trimethoprim/sulfamethoxazole 160 mg every other day, which was prescribed as prophylaxis for pneumocystis pneumonia (PCP) by her pulmonologist. Vitals signs included temperature of 39.1 °C, blood pressure 107/68 mmHg, pulse of 118 beats per minute, respiratory rate of 30 breaths per minute, and oxygen saturation of 90% while on 60 L/min high-flow nasal cannula, with a fraction of inspired oxygen of 0.95. Respiratory requirements during hospitalization are presented in Table 1. Chest examination revealed diffuse coarse crackles bilaterally, with otherwise normal physical examination. Chest X-ray is shown in Fig. 1 and revealed extensive bilateral consolidations. Laboratory tests during hospitalization are presented in Table 2. The patient was shifted to intensive care unit. Polymerase chain reaction (PCR) test for severe acute respiratory syndrome coronavirus 2 (SARS-CoV-2) was positive. Her medications were held except for hydroxychloroquine. Vancomycin 1.36 g daily and meropenem 1000 mg three times daily were started empirically for 4 days then stopped later after negative cultures. Dexamethasone 10 mg IV was commenced for 13 days, along with triple therapy of lopinavir/ritonavir 400/100 mg daily + ribavirin 400 mg twice daily for a total of 14 days. At day 14, her condition improved and she was shifted back to the general ward, with notable improvement in respiratory and biochemical parameters, as illustrated in Tables 1 and 2. Prednisolone was resumed at 15 mg daily to avoid possible flare of connective tissue disease. At day 21, the patient started to be more dyspneic, with repeated vital signs as follows: temperature of 37.2 °C, blood pressure of 110/71 mmHg, pulse of 101 beats per minute, respiratory rate of 28 breaths per minute, and oxygen saturation maintaining 94% using low-flow nasal cannula on 4 L/min. Chest X-ray is shown in Fig. 2, revealing worsening of infiltrates. Repeated labs showed a downward trending of white blood cell (WBC) count from 13,000 to 3900 × 10^9^/L, platelets from 640,000 to 131,000 × 10^9^/L over 7 days, along with increased alanine aminotransferase (ALT) at 138 unit/L (normal 20–65 unit/L) and aspartate transaminase (AST) at 43 unit/L (normal 15–37) unit/L. Repeated C-reactive protein was 51 mg/L. At this point, the clinical and biochemical picture prompted us to widen our differential. Tests for PCP microscopy and staining via induced sputum, serum β-d-glucan, tuberculosis sputum acid-fast bacilli smear and PCR, *Aspergillus* serum galactomannan, varicella, herpes simplex, Epstein–Barr virus (EBV), and CMV were sent. SARS-CoV-2 PCR was repeated as well, which came back negative.

**Table 1:** Respiratory requirements during hospitalization

Days since admission	Oxygen delivery system/support	Oxygen flow rate (L/min)	Respiratory rate	Fraction of inspired oxygen (%)	Oxygen saturation (%)
On admission	High-flow nasal cannula	60	30	95	90
Day 14	Low-flow nasal cannula	2	21	29	98
Day 21	Low-flow nasal cannula	4	28	37	94
Day 39	Ambient air	0	14	21	98

**Table 2:** Laboratory data during hospitalization

Variable	Reference range, adults, this hospital	On admission	Day 14	Day 21	Day 39
Hemoglobin (g/L)	(12.0–16.0)	11.0	10.5	98	108
Hematocrit (%)	(37–47)	33.1	30.8	29.9	32.7
Platelet count (10^9^/L)	(140–450)	460	640	131	350
WBC count (10^9^/L)	(4000–11,000)	5400	13,000	3900	8
Neutrophils (10^9^/L)	(2.0–7.5)	3	4	4	4
Lymphocytes (10^9^/L)	(1.0–5.0)	0.5	4	3	3
Monocytes (10^9^/L)	(0.2–0.8)	0.1	0.5	0.2	0.6
Eosinophils (10^9^/L)	(0.0–0.8)	0.1	0.6	0.1	0.2
Basophils (10^9^/L)	(0.0–0.2)	0.2	0.1	0.1	0.1
Red blood cell count (10^12^/L)	(4.2–5.5)	4	3.8	3.6	0.1
Mean corpuscular volume (fL)	(80–94)	81.9	82	83.2	84
Alanine aminotransferase (unit/L)	(20–65)	90	28	138	40
Aspartate aminotransferase (unit/L)	(15–37)	69	22	43	29
Total bilirubin (μmol/L)	(13–17)	5.78	8.2	15	6
Direct bilirubin (μmol/L)	(0.0–3.00)	2.17	2.0	4	3
Creatinine (mcmol/L)	(53–115)	66	55	60	59
C-reactive protein (mg/L)	(0–10)	20	3	55	0.6

**Fig. 3:**
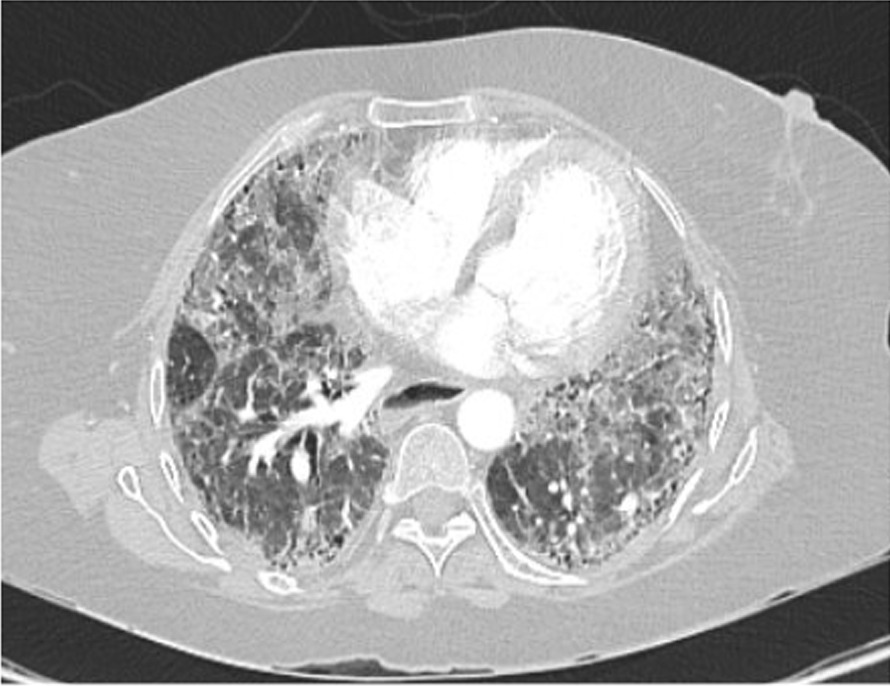
At day 24, chest computed tomography scan showing new bilateral ground-glass opacities scattered in lung field and progression in fibrotic lung changes with interstitial thickening and traction bronchiectasis

**Fig. 4:**
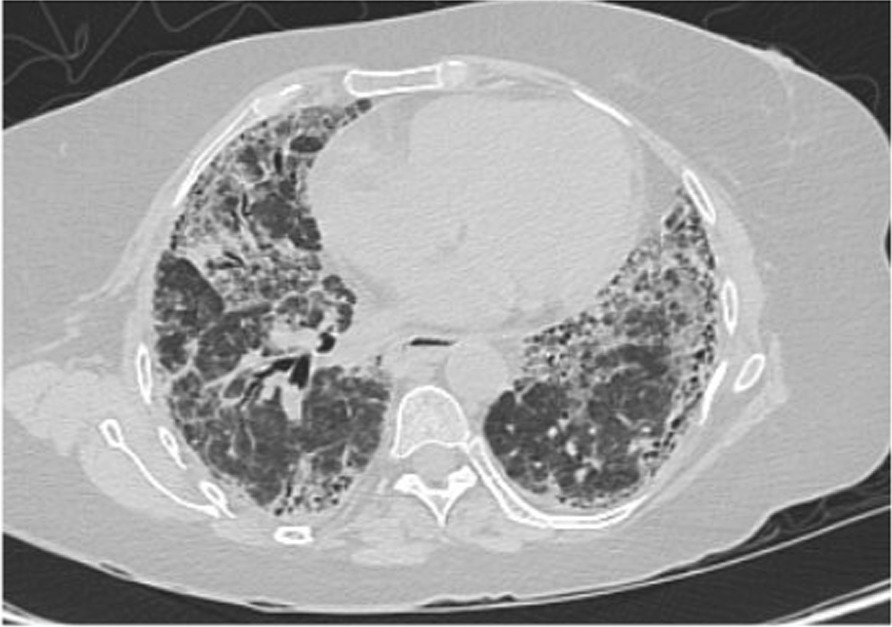
At day 30, high-resolution computed tomography showing interval improvement of bilateral ground-glass opacities with stable fibrotic changes

Vancomycin 1.36 g daily and meropenem 1000 mg three times daily were started empirically to cover hospital-acquired pneumonia. Antibiotics were stopped 2 days later after lack of clinical response, negative cultures, and negative procalcitonin. At day 24, computed tomography (CT) of chest was done and is shown in Fig. 3. As per radiology report, it revealed new bilateral ground-glass opacities scattered in lung field, probably related to infection, as well as mild progression of known UIP in the form of progression in the fibrotic lung changes with interstitial thickening and traction bronchiectasis. However, reassessment following the resolution of the superadded infection should be considered for proper evaluation. Bronchoscopy was not carried out due to hospital precautionary measures in COVID-19 general wards. Test results came back negative for all the aforementioned infections, including PCR for SARS-CoV-2, but positive for CMV IgG and IgM, suggestive of possible CMV reactivation. At day 25, CMV PCR showed 150 copies/ml, which made CMV pneumonitis a quite plausible diagnosis. Based on this, ganciclovir 350 mg IV once daily was commenced. At day 30, high-resolution computed tomography (HRCT) was carried out and is shown in Fig. 4. it revealed interval improvement of bilateral ground-glass opacities with stable fibrotic changes related to UIP, as per report. Ganciclovir was continued to a total of 14 days. At day 39, repeated vital signs showed temperature of 37.0 °C, blood pressure of 115/78 mmHg, pulse of 92 beats per minute, respiratory rate of 14 breaths per minute, and oxygen saturation maintaining 98% at ambient air. Repeated labs were notable for normalized platelet count of 350 × 10^9^/L, C-reactive protein of 0.6 mg/L, and CMV PCR of 20 copies/ml. That patient was discharged on oral valganciclovir 900 twice daily. Four days after discharge, the patient was contacted via phone. Her condition had improved, not requiring oxygen anymore, despite being mildly dyspneic. She continued to feel better each day. She had a follow-up appointment with infectious disease specialist in the ensuing weeks.

## Discussion

Severe COVID-19 is notorious for creating immense immune dysregulation. Release of various acute-phase reactants such as interleukin (IL)-6 has been implicated as one of the mechanism responsible for hypercoagulable state by inducing endothelial damage. Additionally, the possible activation of lectin and alternate complement pathways can induce further endothelial injury [7]. Moreover, such cytokine storm may also favor other infections. Herpesviruses, whose key characteristic feature is persistence following previous exposure, are of mounting concern. In our case, the absence of BAL prompted us to go for less sensitive tests to exclude other infections. The combination of negative sputum microscopy/staining and β-d-glucan for PCP, negative *Aspergillus* serum galactomannan, and negative repeated SARS-CoV-2 PCR was reasonable at that point. However, the diagnosis of CMV pneumonitis in our case was still unequivocal. Although the serum CMV viral load was low, positive CMV IgM suggested reactivation but not particularly CMV end-organ disease. In general, blood testing for CMV DNA was usually found to correlate with BAL findings in confirmed cases of CMV pneumonitis [8]. Since obtaining BAL was not allowed at the time, the concomitant positive CMV IgM, changes in CRP, AST, ALT, platelet counts, changes in chest imaging in a timely manner, along with remarkable clinical, laboratory, and imaging response to ganciclovir made CMV pneumonitis quite suggestive more than any other diagnosis. We believe CMV reactivation or even coinfection with COVID-19 is underrecognized, and increasing numbers of cases are being reported. Marchi *et al*. reported a case of CMV duodenitis and pancreatitis following severe COVID-19 pneumonia, which was successfully treated with ganciclovir [9, 10].

Interestingly, CMV itself may potentate SARS-CoV-2, since they share some innate immunity pathways. Subjects who are carriers of CMV typically show a 30–40% increase in cytotoxic T and natural killer lymphocytes. This causes a reduction in the naïve T cell pool, the primary adaptive immune response in fighting SARS-CoV-2 [11].

In general, CMV reactivation is more common in immunocompromised population than healthy individuals. The population with autoimmune diseases, particularly CTD, carry higher risk, by virtue of impaired immunity, beside concomitant immunosuppressive therapy [12].

The clinical picture was confused by presence of underlying ILD, in which infection can imitate genuine exacerbation. In our case, one idea was to treat as ILD exacerbation. Even if the clinical and radiological pattern are compelling, a mandatory criterion to define exacerbation is to exclude underlying infection. One previously reported case of an SSC patient on mycophenolate mofetil suggests that CMV pneumonitis may mimic the presentation of ILD exacerbation even without a background of ILD [13]. That is indeed a more difficult presentation that highlights the importance of always keeping an open mind regarding the notion of herpesviruses reactivation when dealing with immunosuppressed subjects.

The use of dexamethasone in COVID-19 has yielded the most satisfactory outcome available yet [14]. The current recommendation provided from the RECOVERY trial suggests that low-dose dexamethasone (6 mg) is sufficient [15]. In our case, dexamethasone 10 mg was used, prior to publication of the aforementioned recommendations. In our case, CMV pneumonitis occurred 1 week after dexamethasone course and after achieving remarkable clinical and laboratory improvement, yet steroid use was never completely ceased; she was kept on prednisolone 15 mg to avoid possible ILD flare, besides being hospitalized with and having underlying CTD, putting the patient at probably higher risk compared with other cohorts. When combined with immunosuppressive therapy, the risk of CMV reactivation is indeed high, especially among CTD population. Given that, this may suggest that standardized screening for this population is quite rational.

Among various immunosuppressive therapies that have been tried for cytokine storm, tocilizumab is an anti-interleukin-6 inhibitor that has been introduced as a novel treatment for severe COVID-19 [16]. While results were conflicting, it is fundamental to limit its use on selective bases. Tocilizumab causing CMV reactivation is not uncommon [17, 18]. Its benefit in CTD population and those who are immunosuppressed with severe COVID-19 is highly questionable and might carry a higher risk of reactivation of CMV and other herpesviruses.

## Conclusions

Severe COVID-19 creates immune dysregulation that may favor other infections. Among various opportunistic infections, herpesviruses are of high risk, CMV in particular. Intriguingly, SARS-CoV-2 and CMV may potentiate each other, since they share some innate immune pathways. Subjects with severe COVID-19 and underlying CTD and those who are immunosuppressed carry higher risk compared with other cohorts, which may mandate active surveillance for CMV coinfection. Tocilizumab use in this particular population may carry more harm than previously thought, which may suggest that it is reasonable to omit its use when treating COVID-19.

## Data Availability

All data generated or analyzed during this study are included in this published article.

## Abbreviations

COVID-19: Coronavirus disease 2019
SARS-CoV-2: Severe acute respiratory syndrome coronavirus 2
WHO: World Health Organization
HSV: Herpes simplex virus
CMV: Cytomegalovirus
SLE: Systemic lupus erythematosus
SSC: Systemic sclerosis
ILD: Interstitial lung disease
UIP: Usual interstitial pneumonia
PCP: Pneumocystis pneumonia
PCR: Polymerase chain reaction
EBV: Epstein–Barr virus
WBC: White blood cell
ALT: Alanine aminotransferase
AST: Aspartate transaminase
IgG: Immunoglobulin G
CT: Computed tomography
HRCT: High-resolution computed tomography
